# Bilateral Parainfectious Optic Neuritis in Young Patient

**DOI:** 10.7759/cureus.29220

**Published:** 2022-09-16

**Authors:** Sruban Suparmaniam, Wan-Hazabbah Wan Hitam, Saritrasaraswathy Thilagaraj

**Affiliations:** 1 Department of Ophthalmology and Visual Sciences, School of Medical Sciences, Health Campus, Universiti Sains Malaysia, Kelantan, MYS; 2 Department of Ophthalmology, Hospital Sultanah Bahiyah, Kedah, MYS

**Keywords:** pediatric optic neuritis, blindness, meningitis, optic neuritis, parainfectious

## Abstract

Parainfectious optic neuritis arises from infectious aetiology either from pathogen direct invasion or after an infectious disease which can be immunologically mediated demyelination of optic nerve or, from inflammation of optic disc vasculature. We report a case of bilateral optic neuritis in a young patient. A 13-year-old boy presented with painless profound vision loss in both eyes preceded by an episode of fever two weeks prior. Visual acuity in both eyes was a perception of light. Fundoscopy showed a bilateral hyperemic swollen disc. Blood investigations were normal except for C-reactive protein and ESR was elevated. CSF analysis was also normal with no growth of micro-organisms. Both CT scans and MRIs of the brain and orbit showed normal findings. The patient was diagnosed to have parainfectious optic neuritis. He was started on intravenous methylprednisolone for five days followed by a tapering dose of oral prednisolone for a total of one month. His final visual acuity improved to 6/6 in both eyes with a normal optic disc appearance.

## Introduction

Optic neuritis is an inflammatory optic neuropathy that causes visual impairment and is associated with demyelinating inflammatory disease and infectious aetiologies. It typically affects patients between 15 and 45 years old. It is a clinical diagnosis based on a patient's history and clinical findings. It may occur at any age, but lower incidences are found in children with an annual incidence of optic neuritis being 1.04 per 100 000 people (Korean population) with slightly female predilection [1]. Parainfectious optic neuritis is known as optic neuritis arising from infectious aetiology that develops days to weeks after infection. It can be either from pathogen direct invasion or after an infectious disease-causing immunologically mediated demyelination of the optic nerve or from inflammation of optic disc vasculature [2]. We report a rare case of bilateral optic neuritis in children secondary to parainfectious.

## Case presentation

A 13-year-old previously healthy boy presented to the ophthalmology clinic with acute painless profound vision loss in both eyes for three days. It initially started in the right eye (RE) and a day later involved the left eye (LE). The vision loss started in the central area and then progressively became generalised. It was preceded by episodes of low-grade fever and headache two weeks prior. He did not seek any treatment. There was no history of somnolence, altered consciousness, or limb weakness. He also did not have any nausea, vomiting, chronic cough, or rashes. 

**Figure 1:**
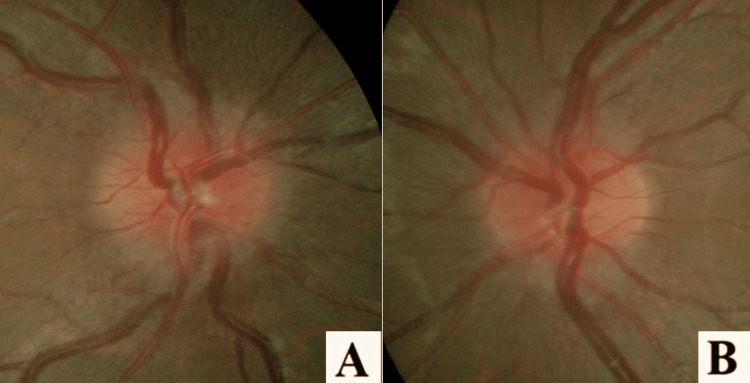
(A, B) Both eyes optic disc (OD) swollen and hyperemic with the right eye being more prominent. Both maculae were normal.

On general examination, his vital signs were normal but obese with his body mass index (BMI) of 35.9 kg/m^2^ (weight: 92kg, height 1.6m). Systemic and neurological examinations were normal. His visual acuity was only a perception of light in both eyes with sluggish pupillary light reflex. Extraocular muscle movements were normal. Anterior segment examination was unremarkable in both eyes. Fundoscopy (Figures 1A, 1B) of the right eye showed swollen and hyperaemic disc. Spontaneous venous pulsations were present and there were no signs of nerve fibre layer infarct. The left optic disc was less swollen and hyperemic. The macula on both sides was normal. There were no signs of vitritis, vasculitis, or retinitis.

His significant blood results and CSF analysis that shows lymphocytic pleocytosis are shown in Table 1. Meanwhile his haemoglobin level and total white blood cell count were normal. Serological infectious tests for toxoplasmosis, syphilis, leptospirosis, mycoplasma, HIV, hepatitis C virus, hepatitis B virus and herpes-simplex virus were normal. Connective tissue screening for antinuclear antibodies (ANA), perinuclear antineutrophil cytoplasmic antibodies (p-ANCAs), cytoplasmic ANCAs (c-ANCAs), antiaquaporin 4 (AQ4) antibodies and myelin oligodendrocyte glycoprotein (MOG) antibody was also negative. An urgent CT scan of the brain and orbit revealed normal findings. MRI showed a normal optic nerve appearance without any other brain lesion.

**Table 1:** Investigation results CRP = C-Reactive Protein, ESR = Erythrocyte Sedimentation Rate, CSF = Cerebrospinal fluid

Investigation	Result	Unit	Reference range
CRP	14	mg/L	<8
ESR	99	mm/hr	10-14
CSF: opening pressure	15	cm H_2_O	5-25 (in obese)
CSF: Cell count	250 (90% Lymphocytes)	mm^3^	0-10
CSF: Glucose	55	mg/dL	50-80
CSF: Protein	38	mg/dL	20-40
CSF: Lactate	1.79	mg/dL	1-3
CSF: Culture	No organism		

**Figure 2:**
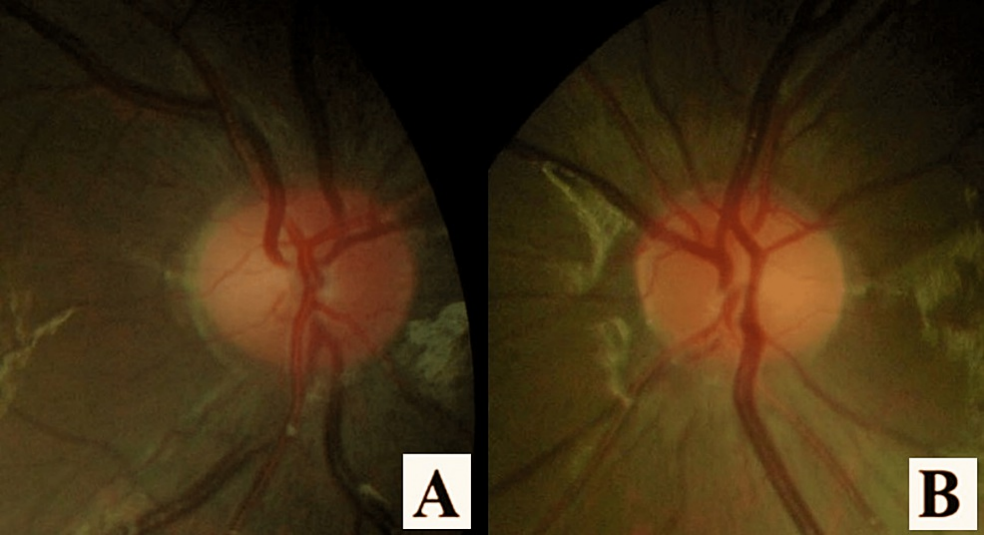
(A, B) Resolving OD swelling after three weeks

The patient was diagnosed to have bilateral optic neuritis secondary to parainfection. He was started on a course of intravenous methylprednisolone 250mg four times a day (total dose 1g/day) for five days, followed by oral prednisolone 60mg once daily for nine days. The oral prednisolone was tapered over two weeks. After one week of corticosteroid, his best-corrected vision improved to 6/36 in the right eye and 6/18 in the left eye. Optic disc swelling in both eyes resolved in three weeks and both visual acuities improved to 6/6. (Figures 2A, 2B). On follow up, visual acuity and fundus in both eyes remained the same.

## Discussion

Optic neuritis can be associated with an infectious cause, parainfectious process, CNS demyelinating condition, or not associated with any disease [2]. A paediatric optic neuritis is an atypical form that usually features bilateral papillitis. The general comparison of paediatric optic neuritis cases in Malaysia shows a higher occurrence in females [3]. Optic neuritis in Malaysia shows 50% of aetiology related to infection [4]. Parainfectious optic neuritis is defined as the involvement of the optic nerve that occurs after presumably or confirmed systemic infectious disease and is highly associated with viral aetiology [5]. Gender predilection in parainfectious optic neuritis is seen more in female predominance, especially after puberty. Visual acuity appeared to be worse in children compared to adults, but the precise epidemiological characterisation is not feasible due to its rarity and to date. There is limited information about this entity in the literature [2].

Typically, parainfectious optic neuritis follows the primary disease within one to three weeks. The patient presents with prodromal systemic symptoms, but they can be subtle [6]. Although the pathogenesis of this disease is still uncertain, the theory of parainfectious optic neuritis is due to an immunologic-inflammatory reaction supported by the diagnosis of optic neuritis days to weeks after the presumed systemic infection [2]. Visual acuity on presentation tended to be worse in children compared to adults but had equally good visual outcomes and all patients had swollen discs [2]. In paediatric patients, the time elapsed between the febrile illness and the onset of the visual symptoms was shorter. It can be explained by a more fulminant immune response in children, with bilateral involvement of the optic nerve and illness may affect other parts of the nervous system (e.g., cerebellitis or meningoencephalitis) [6].

The diagnostic challenge of bilateral optic disc swelling in the paediatrics group is due to the multitude of aetiologies (infectious, autoimmune, neurologic, vascular, and genetic). In this case, papilloedema and pseudopapilloedema were excluded, the first by radiological imaging and the latter by optic disc imaging. In the absence of comorbidities and considering his age. The vascular cause is excluded together with both arthritic and non-arthritic optic neuropathies as a possible diagnosis. Genetic cause such as Leber's hereditary optic neuropathy (LHON) was also considered but usually, the disease occurs between the second and fourth decades, with a subacute presentation and did not reveal the presence of OD appearance presence of telangiectatic microangiopathy at the optic disc.

Parainfectious optic neuritis can occur at any age and is usually caused by pathogens such as mycoplasma pneumonia, Varicella-zoster virus (VZV), rubella, mumps, and rarely, Epstein-Barr virus (EBV) [7]. The most frequent cause is a viral infection, but it is not always possible to identify the causal organism [5]. Extension from adjacent meningitis or orbital disease can cause direct infection of the optic nerve. This situation is not always obvious clinically, however, the patient outcome can be critically influenced by early consideration of unusual organisms [6]. A retrospective case series identified that 67% of the main pathogen in the children's group was Mycoplasma pneumoniae. However, in this case, the serum anti-mycoplasma was negative. Neurological complications of M. pneumoniae infection in children include encephalitis and half of them had bilateral eye involvement [2]. M. pneumoniae-associated optic neuritis is commonly seen in children and young adults and should be taken into consideration in cases of isolated optic neuritis despite the patient does not exhibit any signs of respiratory infection [8]. Parainfectious optic neuritis can be related to ADEM and encephalomyelitis [9]. Unlike, in this case, it is reported about 40% of cases had MRI findings consistent with ADEM [2]. There has also been reported parainfectious optic neuritis related to COVID-19 [10]. However, this case was presented in 2019 before the COVID-19 pandemic.

There have been no clinical trials in children for the treatment of optic neuritis in contrast to the optic neuritis treatment trial (ONTT) study in adults. Moreover, there is no consensus regarding therapy for parainfectious optic neuritis [7]. Intravenous methylprednisolone pulse therapy is known to have a good visual prognosis, with or without antiviral coverage as well [5]. Among authors, the widest recommendation; cases of unilateral paediatric optic neuritis are conservative, while for bilateral with severe vision loss are to be treated with a short dosage of intravenous methylprednisolone (15 mg/kg/day for three days). Plasma exchange has been proposed as another treatment modality to achieve full recovery of vision for cases of steroid-resistant bilateral optic neuritis [11]. In this case, the patient received a high dose of intravenous methylprednisolone with antibiotics and antiviral as well. Then continued with oral prednisolone and tapered over the course for another two weeks (in a total of four weeks).

Although generally, the visual recovery is good, based on the case series the treatment was not associated with improved outcomes in terms of visual acuity, visual fields, or residual optic nerve damage. During the follow-up period within six months, there were no episodes of recurrent optic neuritis [2]. In this patient, his visual acuity recovered to 6/6 for both eyes, but in terms of outcome, we were unable to access him further as he defaulted subsequent follow-up.

Parainfectious optic neuritis occurs after presumably or confirmed systemic infectious disease (frequently viral). This is a very difficult area without absolute guidelines, but intravenous corticosteroids are the most frequent option with good visual recovery. A high index of suspicion is required among physicians with further investigation and reports required to elucidate disease pathogenesis and to guide proper management.

## Conclusions

Optic neuritis is uncommon in the paediatric population. Parainfectious optic neuritis is rare in nature and due to limited documentation regarding this disease, it makes as a diagnosis of exclusion. Clues to the fact that prodromal systemic symptoms need to be considered and be more vigilant if involving the immunocompromised patient. Treatment tendency, especially in cases of bilateral involvement with severe vision loss, recommends short dosages of intravenous corticosteroids. The visual prognosis is generally good regardless of age.

## References

[1] Lock JH, Newman NJ, Biousse V, Peragallo JH (2019). Update on pediatric optic neuritis. Curr Opin Ophthalmol.

[2] Rappoport D, Goldenberg-Cohen N, Luckman J, Leiba H (2014). Parainfectious optic neuritis: manifestations in children vs adults. J Neuroophthalmol.

[3] Shatriah I, Hitam WHW, Nor-Idahriani MN, Jakiyah D, Zunaina E (2012). Clinical profile and aetiology of optic neuritis in hospital Universiti Sains Malaysia-5 years review. Med J Malaysia.

[4] Shatriah I, Adlina AR, Alshaarawi S, Wan-Hitam WH (2012). Clinical profile of Malay children with optic neuritis. Pediatr Neurol.

[5] Hipolito-Fernandes D, Elisa-Luís M, Trigo M, Tavares-Ferreira J (2019). Parainfectious optic neuritis followed by microcystic macular oedema. BMJ Case Rep.

[6] Weerasinghe D, Lueck C (2016). Mimics and chameleons of optic neuritis. Pract Neurol.

[7] Shima T, Nagaoka A, Yoshimura S, Oka A, Matsumoto M, Kitaoka T, Tsujino A (2020). An adult case of parainfectious optic neuritis associated with genital herpes simplex virus type 2 infection. Clin Neurol Neurosurg.

[8] Celi̇k H, Aksoy e, Öztoprak Ü, Akçaboy M, Uysal Yazi̇ci̇ M, Savas Sen Z, Yüksel D (2022). Neurological manifestations of Mycoplasma pneumoniae infection in hospitalized children: a single-center experience. Turkish J Pediatr Dis.

[9] Gise RA, Heidary G (2020). Update on pediatric optic neuritis. Curr Neurol Neurosci Rep.

[10] Fernández Alcalde C, Granados Fernández M, Nieves Moreno M, Calvo Rey C, Falces Romero I, Noval Martín S (2021). COVID-19 ocular findings in children: a case series. World J Pediatr.

[11] Pérez-Cambrodí RJ, Gómez-Hurtado Cubillana A, Merino-Suárez ML, Piñero-Llorens DP, Laria-Ochaita C (2014). Optic neuritis in pediatric population: a review in current tendencies of diagnosis and management. J Optom.

